# Climate Change and Companion Animals: Identifying Links and Opportunities for Mitigation and Adaptation Strategies

**DOI:** 10.1093/icb/icab025

**Published:** 2021-04-19

**Authors:** Alexandra Protopopova, Lexis H Ly, Bailey H Eagan, Kelsea M Brown

**Affiliations:** 1 Faculty of Land and Food Systems, Animal Welfare Program, University of British Columbia, Vancouver, British Columbia, Canada; 2 Independent Research Scientist, Chicago, IL, USA

## Abstract

Recent natural disasters and weather extremes are a stark reminder that we live in a climate crisis. Climate scientists and policymakers have asked each discipline to anticipate and create mitigation and adaptation plans in preparation for a worsening future. Companion animals both impact and are impacted by the changing climate through their intrinsically linked relationships to human society. In this theoretical paper, we argue that companion animal scientists are well-suited to address climate change issues. We identify several anticipated climate change outcomes, such as an increase in extreme weather events, human migration, disasters, and an increase in human inequity, and connect these outcomes to identified or hypothesized impacts on companion animals and the human–animal bond. We suggest opportunities to reduce climate change impacts on companion animals that include alterations to owner caretaking behaviors and breeding practices, and education of owners and governments on zoonosis and disaster preparedness. Furthermore, building climate resilience through decreasing inequity in companion animal fields is paramount; and we propose that a starting place can be in animal sheltering and other support services. We also summarize how companion animals and owners’ caretaking behaviors are impacting climate change through the use of finite natural resources as well as pollution and carbon emissions. We propose that replacement, reduction, and refinement, that guide laboratory animal research, can also be useful to mitigate the effects of companion animals on the environment. We suggest criteria for successful mitigation and adaptation plans to include equitability, sustainability, respect for animals, and measurability. Finally, we end on a call to all companion animal professionals to actively consider their role in mitigating the impact of companion animals on the climate and preparing for the fallout of climate change in their communities.

## Introduction

On April 22, 2016—Earth Day—nearly 200 nations worldwide signed the Paris Agreement within the United Nations Framework Convention on Climate Change (IPCC 2014). Since then, countries have implemented strategies to reduce greenhouse gas (GHG) emissions and allocated funds and resources to combating climate change (Eskander and Fankhauser 2020). However, the burden to act does not fall solely on federal, state, and provincial governments. As scientists, we can advance the Paris Agreement’s goals in our own scientific and applied fields by examining how we may lessen the current impact of our study population on the climate (“mitigation strategies”) and how we may lessen the harmful effects of impending climate outcomes on our study population (“adaptation strategies”; Grafakos et al. 2019). The intersection of global climate change and companion animal–human interaction, and its application, has yet to be seriously addressed (Stephen et al. 2019).

### Projected impacts of climate change

The World Health Organization (WHO) has declared climate change to be the most significant threat to public health (World Health Organization 2019). A devastating collection of global consequences have been forecast; scientists anticipate necessary costly adaptations due to rising sea levels, forced displacement, and migration (Marchiori and Schumacher 2011; Hoffmann et al. 2020), shrinking productivity of harvests, widespread poverty (Leichenko and Silva 2014), new and increasing diseases (Kalkstein and Smoyer 1993; c.f., Rohr et al. 2011; Davidson et al. 2012; Otranto et al. 2017), freshwater shortages, increased warfare (Zhang et al. 2007a, 2007b), social injustices, and depletion of natural resources (Martens et al. 2019).

Scientists predict that climate change will further escalate the frequency and intensity of extreme weather events, leading to more disasters disrupting communities globally (Banholzer et al. 2014). For example, as a result of melting land ice and the expansion of warmer seawater, global sea level is expected to rise by 0.3–2.4 m by 2100, leading to increased flooding due to storm surges and high tides (Church and White 2011). Hurricanes in the North Atlantic are projected to become more intense and higher in frequency and duration (Bhatia et al. 2019). Higher temperatures are increasing the size and occurrence of wildfires, as well as lengthening the wildfire season in many areas (Cassell et al. 2019). By the end of the century, extreme heat events are expected to occur every few years (NASA, 2021).

A particularly cruel aspect of climate change is that the negative impacts will disproportionately affect those who are already marginalized: people of color and lower-income communities (B Corp Climate Collective 2021). At the same time, social injustices are expected to *increase* in the wake of climate change (Islam and Winkel 2017; Diffenbaugh and Burke 2019). Existing racial and economic inequities will be exacerbated by mass migration of populations (Marchiori and Schumacher 2011; Hoffmann et al. 2020), competition for increasingly limited resources (Wheeler and Von Braun 2013; Janssens et al. 2020), and growing political unrest (Zhang et al. 2007a; Nardulli et al. 2015). Thus, responding to and preventing further social injustices is a crucial component of comprehensive climate change mitigation and adaptation strategies.

### Companion animals are relevant to climate change discussions

Companion animals play a significant role in society and the world economy. While global estimates of companion animals are unknown, in 2018 over 373 million cats and 471 million dogs were estimated to be kept as companion animals worldwide (Euromonitor 2019, as cited in Sivewright and Kreuger 2019). This is likely a considerable underestimation due to the number of unregistered animals, other companion animal species not being accounted for (e.g., birds, reptiles, and small mammals), and the continual rise of companion animal ownership (Martens et al. 2019). The number of free-roaming domestic dogs worldwide is an estimated 700 million to 1 billion (Hughes and Macdonald 2013; Gompper 2014 as cited in Sykes et al. 2020), and worldwide free-roaming cat population estimates are over 480 million (CAROcat 2021).

People spend monetary and natural resources on their companion animals. The global pet care and supplies market is ∼225 billion USD and is expected to increase to 359 billion by 2027 (GlobeNewswire 2020). In addition, people incorporate their pets’ needs into daily decisions. A study of elderly, low-income residents of the United States reported that 8% would stay at home with their pets during an emergency evacuation, with another 16% stating they would not leave without their pets (Rosenkoetter et al. 2007). More recently, Applebaum et al. (2020) found that >10% of survey participants said they might delay or avoid treatment of COVID-19 due to concerns for their pet’s welfare. Because of this intense connection with humans, companion animals as well as the human–animal bond are directly and indirectly affected by global issues (Sykes et al. 2020), such as the consequences of climate change.

Furthermore, companion animals and their care requirements likely contribute to climate change. As many households consider pets as family members, with some even reporting feeling closer to their pets than human family members (Barker and Barker 1988; Walsh 2009a), owners spend substantial time and resources ensuring care for their companion animals. As such, owner behaviors have direct impacts on climate change. Production of companion animal food is considered a “neglected predictor of environmental damage” (Su et al. 2018). Okin (2017) estimated that, in the United States, cat and dog diets account for ∼30% of the environmental impacts of food production; however, on a global scale, these impacts are perhaps lower. While companion animal food was previously restricted to nonhuman-grade and waste products, a trend of “humanization” of pet food is occurring (Clemens 2014). Many guardians now prefer human-grade ingredients with high-animal protein content and may feed an excess of nutrients, further increasing the environmental impact (Swanson et al. 2013). In fact, shifting preferences currently make companion animal feed a direct competitor to human food consumption (Martens et al. 2019); a notable concern considering that climate change is increasing global food insecurity (FAO, IFAD, UNICEF, WFP, and WHO 2020).

Despite these connections, companion animal scientists and practitioners have been relatively silent on issues of climate change. An anecdotal review of various veterinary associations revealed that none identified climate change as a priority area (Stephen et al. 2019). At the same time, veterinary professionals reported enthusiasm for taking leadership roles in climate change topics, but this enthusiasm was curtailed by a lack of educational resources (Kramer et al. 2020; Pollard et al. 2020). A recent call to action aimed at veterinarians provided a framework for situating the veterinary community to combat climate change. The identified connections included promoting animal health as part of climate change plans, communicating the implications of climate change on various animal populations, educating lawmakers on animal health, including climate change curriculum in veterinary education, among several others (Stephen et al. 2019). However, veterinarians are not the only ones who can make an impact.

### Companion animal scientists are ideally suited to contribute to climate issues

Scientists who study companion animal–human interactions are also well-suited to contribute to conversations regarding climate change. Working toward sustainable climate mitigation and adaptation strategies requires us to engage in whole-systems thinking rather than focus on individual components (Stephen 2021). Strengthening the ability for researchers to be “specialized generalists” that are capable of bringing both specialized knowledge of their field as well as a significant capacity to collaborate with other fields is necessary for climate resiliency research (Parkes 2021). Companion animal scientists are already “specialized generalists,” with most studying both biological sciences as well as the social aspects of the human–animal relationship. Researchers who study companion animals have backgrounds in many different kinds of disciplines, ranging from animal and veterinary science (e.g., Van Haaften et al. 2017), psychology (e.g., Wynne 2016), biology (e.g., Lindblad-Toh et al. 2005), anthropology (e.g., MacLean et al. 2017), public health (e.g., Bauman et al. 2001), nursing (e.g., Banks et al. 2008), and even archeology and biogeography (e.g., Larson et al. 2012), among many others. And many companion animal focused conferences bring together differing disciplines. In fact, companion animal scientists are embracing the One Health and/or One Welfare framework, which recognizes the connection between humans, animals, and the environment (Sykes et al. 2020). This interdisciplinary and holistic framework identifies that multiple systems are intrinsically linked and interventions must be understood as part of a whole—wherein improving the health/well-being of one of the three facets ultimately improves the others (Pinillos et al. 2016). As such, companion animal scientists may already be experienced in systems thinking and collaborations across disciplines in their work, positioning them well for incorporating climate change complexities into their research.

Furthermore, many researchers within the field of human–animal interaction work in close collaboration with their population communities conducting applied research and extension work, such as directly advising pet owners and members of the pet industry (dog trainers, animal shelter staff, pet food companies, etc.). Industry conferences invite scientific as well as industry perspectives. Therefore, companion animal scientists are in an ideal position to implement direct changes in current practices. Given that the translation and broad implementation of newly discovered scientific knowledge takes a substantial amount of time (Morris et al. 2011), already established working relationships between companion animal scientists and companion animal stakeholders are likely to reduce research-to-implementation time lags. Given the urgency of climate change, these already established relationships are a significant benefit.

## Authors’ objectives

In this article, we follow the recommendations set forth in the Paris Agreement by first identifying how the impact of climate change directly and indirectly affects companion animals and the human–animal bond, and identifying how companion animals contribute to climate change. Second, we identify opportunities for mitigation and adaptation strategies to both prepare for the fallout of climate change as well as lessen the negative impacts of companion animals on climate change. Finally, we highlight features of successful and just adaptation plans as outlined by social scientists and end on a call to all companion animal professionals to actively consider their role in mitigating the impact of companion animals on the climate and preparing to adapt to the fallout of climate change.

## Impact of climate change on companion animals

### Extreme weather events and weather changes

Increasing warm weather—an outcome predicted to occur with high likelihood—has a direct impact on companion animal health. Several animal welfare organizations have already reported that incidences of dogs dying in hot cars are increasing (British Veterinary Association 2019; Shih et al. 2019). Increase in warm temperature also has an alarming correlation with an increase in dog bites (Ahmed et al. 2019) and rabies infections in dogs (Lachica et al. 2020). An increase in droughts may also worsen the rabies epidemic as rabies infection has been negatively correlated with precipitation (Courtin et al. 2000; Lachica et al. 2020).

One global impact of climate change is the increased spread of infectious diseases, including vector borne and zoonotic diseases (Mills et al. 2010). Climate change has allowed for the geographic range of vector species to expand or shift, thus exposing greater animal and human populations to diseases, as well as unknown emerging zoonoses (Mills et al. 2010). Similarly, climate change impacts the carrying capacity of ecosystems, which can alter the population density of host or vector species (Mills et al. 2010). Increasing warm weather has already been found to increase the prevalence of ticks and tick-borne illnesses such as Lyme disease (Lindgren and Gustafson 2001; Gray et al. 2009; Estrada-Peña et al. 2012). While Canada’s southern border was previously the northern extreme of the tick’s geographic range, surveillance of Lyme disease in humans and animals shows that climate warming contributes to the emergence of Lyme disease beyond previously known ranges (Ogden et al. 2019). Heartworm infections have also been found to vary according to climate and weather patterns (McGill et al. 2019; Széll et al. 2020).

A further harm may come from people reacting to a changing climate by changing their animal caretaking behaviors. An increase in hot weather and extreme weather events may lead to a reduction of exercise opportunities for companion dogs (Schneider et al. 2015), which may further exacerbate pet obesity (Hurley et al. 2011). As dog walking and other mutual activities improve the human–dog bond and may protect against behavioral problems, such as barking, hyperactivity, and aggression (Kobelt et al. 2003; Bennett and Rohlf 2007; Curb et al. 2013), a decrease in walking opportunities may endanger the bond and lead to increased relinquishment of dogs to shelters.

The frost-free season’s length is projected to increase by at least 1 month by the year 2100 across the United States (NASA, 2021). Anecdotally, animal shelters are concerned that longer summers mean a longer kitten season by increasing kitten survival rates, decreasing latency to sexual maturity, or increasing prey abundance (Arlington 2016). This may further worsen the urgent issue of cat overpopulation. Moreover, researchers have predicted that free-roaming cat populations will increase as human emissions increase—a likely outcome as human populations continue to rise (Aguilar et al. 2015).

### Human migration and companion animals

The Intergovernmental Panel on Climate Change (IPCC) warns that the most significant single impact of climate change will be in human migration events as humans will outnumber their local environments’ carrying capacities. Typical estimates report that 200 million people are expected to become “climate refugees” by 2050 (nearly 3% of the world population, c.f. Brown 2008). As companion animals go where people go, we can also assume a higher proportion of companion animals will also be migrating with their owners. Long-distance transport is a direct stressor for companion animals. Dogs show behavioral and physiological markers of stress during ground and air transport (Bergeron et al. 2002). Cats are likely at an even higher risk of substantial distress as they show an acute stress response even to short-distance travel between the home and a veterinary clinic (Nibblett et al. 2015). Imported and transported animals may also bring “exotic” zoonotic diseases that are not endemic at destination communities (Anderson et al. 2019; Polak 2019). For example, an increased movement of dogs, albeit shelter not pet dogs, from southern to northern Europe resulted in the introduction of new parasites (Otranto et al. 2017). Further, rabies is domestically controlled across many countries, but imported dogs may pose serious risk of introducing foreign variants, which can directly affect human health and cause the establishment of novel canine rabies strains (McQuiston et al. 2008).

On the other hand, people may elect to leave their companion animals behind as they are forced to move. Pet abandonment during evacuation is a commonly reported issue in disasters (Edmonds and Cutter 2008). Pet evacuation failure is related to the human–animal bond—pet owners with lower attachment and commitment scores are more likely to evacuate without their pets (Heath et al. 2001). Pet owners report many reasons for leaving their animals behind, including believing they will return to the animal shortly, being away from home at the time of disaster, or being unable to take animals to emergency accommodations (Walsh 2009a; Chur‐Hansen 2010). A leading cause of surrender of companion animals into shelters is moving (Coe et al. 2014; Lambert et al. 2015; Jensen et al. 2020). Owned and unowned animals left behind during emergencies may pose additional health risks to humans and animals, including increased transmission of zoonotic disease, contamination of water and food, and increased aggression toward other animals and humans due to fear (Leonard and Scammon 2007; Travis 2014).

### Opportunities to reduce climate change impact on companion animals

Preserving the health of companion animals in a warming climate involves alterations in both owner behaviors as well as breeding practices. Aside from educating owners on the increased risks of leaving their pets in hot cars, more indirect opportunities may exist to safeguard animals. For example, obesity plays a key role in heat tolerance (Hall et al. 2020). Unfortunately, an estimate of 60% of companion animals in the United States are overweight or obese (APOP 2019) and are thus at a higher risk for heat-related medical concerns. Furthermore, whereas brachycephalic breeds, such as French bulldogs and Chow Chows, are highly popular, they are also at a higher risk for heatstroke (Davis et al. 2017; Hall et al. 2020). These risk factors are another reason to control the pet obesity epidemic and improve breeding practices to avoid phenotypes such as brachycephaly, which directly cause health and welfare concerns for individual animals (Ladlow et al. 2018; Harvey et al. 2019; Packer et al. 2019; Hall et al. 2020).

Increasing ranges of parasites and infectious diseases threaten both animal and human health, and researchers have identified the role of both veterinary and medical practitioners in educating the public about related risks (Damborg et al. 2016). However, companion animal research is lacking in the area of emerging zoonoses in comparison to other facets of animal use. For example, there are global surveillance programs in place to track the emergence and prevalence of zoonoses in wildlife and livestock; the same surveillance measures in companion animals will likely be needed (Day et al. 2012; Goni et al. 2018). The One Health paradigm emphasizes that the majority of emerging infectious diseases in humans will derive from animal reservoirs, although the focus of this pathway is mainly on livestock and wildlife (Overgaauw et al. 2020). However, companion animals can also serve as possible sources of zoonotic infections (Halliday et al. 2007). Pet ownership is a unique risk for zoonotic disease transfer due to the consistently close proximity of pets to humans. Over time, more dogs and cats are spending increased time indoors with their owners (Day et al. 2012), which may both increase risk to humans due to increased proximity, but also decrease risk due to increased vaccinations and sanitation. Because zoonosis falls at a distinctive intersection of human and animal health, it is crucial to understand the role of companion animals in this domain.

Whereas an increase in heatstroke and increased risk of disease in companion animals has already been identified as a current outcome of the changing climate, other impacts are hypothesized. Therefore, opportunities may include further research into owner caretaking behaviors during increasingly warm weather and the relationship between pet relinquishment, human migration events, and the changing climate. Identifying interventions to reduce transport-associated stress in companion animals, such as the use of anxiolytic medications (e.g., Van Haaften et al. 2017), will likely be essential.

We have identified a few confirmed and hypothesized impacts on companion animal health; however, there are certainly many more opportunities that exist to prepare and reduce the harm of the changing climate on companion animals. The next section will draw several more links between the climate outcomes and harms to the human–companion animal bond.

## Impact of climate change on the human–animal bond

### Disasters and companion animals

Disasters such as wildfires, hurricanes, and flooding are likely to increase in frequency (Banholzer et al. 2014). The strength of the human–animal bond is particularly evident during times of disaster, where pet owners often choose to risk their own lives to save animals from fires, floods, and hurricanes (Thompson 2013). Even though previous events outline the importance of pet-related planning during disasters, very few governments’ disaster plans incorporate companion animals. For pet owners, dealing with disasters is contingent on decision making about not only themselves, but also the animals in their households (Travers et al. 2017). Research shows that pet ownership increases risk of evacuation noncompliance and unauthorized early re-entry attempts. Evacuation noncompliance not only creates risks to both the pet owner and the animal, but also to emergency response teams in the community (Chadwin 2017). Difficult decisions during emergencies and natural disasters elevate risk for pet owners, their animals, and the community, indicating that the human–animal bond should not be trivialized when planning for these events.

Forced separation from pets can negatively impact the mental health of pet owners. Losing a pet in the case of natural disaster leads to significant grief and mourning (Hunt et al. 2008). Pet loss increases risk of post-traumatic stress and intensifies the severity of depressive symptoms following a disaster (Hall et al. 2004; Lowe et al. 2009). Finally, families that are separated from companion animals during disasters face further challenges when attempting to find their pets—and reunion rates remain low (Heath and Linnabary 2015).

### Opportunities for preserving the human–animal bond during disasters

In response to previous major disasters, new measures have been introduced to protect companion animals and owners in the future: because of the fallout from Hurricane Katrina, the Pet Evacuation and Transportation Standards Act in the United States now requires provisioning of pet-friendly shelters after a disaster (Reed et al. 2020). However, research on pet-related disaster planning is mainly limited to the Anglosphere, and research and policy implementation are still needed globally. For example, approximately 10,000 owned dogs were reported dead after the 2011 earthquakes in Japan (Yamazaki 2015). Furthermore, there must be greater considerations for companion animals during disasters in at risk areas, such as those with greater vulnerable human and animal populations. For instance, the Philippines, a highly disaster-prone country due to its location, also has a high population of free ranging dogs, which could place further risks to both humans and animals during times of disaster (Davlin et al. 2013; De Leon and Pittock 2017). And finally, countries around the world will have to consider their distinctive natural and social environments, as communities will ultimately be impacted differently by the increasing threat of disasters due to climate change.

Although pet ownership creates an increased risk during disasters, companion animals simultaneously increase resilience for humans (Thompson et al. 2014). Companion animals reduce distress associated with traumatic events, and the human–animal bond can act as a strong support following disasters (Thompson 2013). The strength of the human–companion animal bond therefore warrants proper consideration during disaster preparation and management. Continued research on the human–companion animal relationship during crises will certainly serve to better prepare for impacts of future pandemics and other disasters.

### Human vulnerabilities and inequities

Emergency decisions during disasters are shaped by external forces, meaning that the effects of disasters on pet owners are even greater when coupled with other vulnerabilities. Structural factors such as community disaster preparedness, proximity to human and animal emergency services, availability of transportation, and knowledge about disasters can impact responses (Every et al. 2016). On top of that, social factors such as age, race/ethnicity, and socioeconomic status can contribute to further vulnerability during and following disasters (Thompson et al. 2014). For example, older adults are less likely to have the ability to drive, which decreases the likelihood of being able to access nearby animal shelters (Douglas et al. 2019). Minority and immigrant populations are more vulnerable to disasters due to lack of access to disaster-related information in a native language, including those pertaining to pet evacuation (Mathew and Kelly 2008). Individuals experiencing poverty may face additional financial barriers, as some pet-friendly evacuation shelters require pets to be recently vaccinated prior to entry (Douglas et al. 2019). Some may not own sufficient carriers for their pets to evacuate safely, which has been identified as a significant hindrance to the evacuation process (Heath et al. 2001). Following disasters, pet owners who live in rental dwellings face serious difficulties finding pet-friendly rentals, and may end up needing to relinquish their animals or live in unsafe dwellings to keep their pets (Coe et al. 2014; Graham and Rock 2019).

With increased competition for natural resources, migration of human populations across the globe, and growing political unrest (Zhang et al. 2007a; Marchiori and Schumacher 2011; Wheeler and Von Braun 2013; Nardulli et al. 2015; Hoffmann et al. 2020; Janssens et al. 2020), the resulting expected increase in human inequities and injustices will doubtless affect companion animal ownership. Veterinarians report that access to veterinary care is one of the main challenges for pet health in North America (LaVallee et al. 2017; Harding 2018). Individuals of lower socioeconomic status are less likely to have regular visits to their veterinarians and experience pet care deserts (Fung et al. 2014; Spencer et al. 2017; Tran et al. 2019). Likewise, human vulnerabilities predicted increased risk of surrendering animals for owner-related reasons and surrendering animals that were not considered healthy upon intake (Ly et al. 2021). Furthermore, whereas most diseases affecting companion animals are not commonly transmitted to healthy humans, immunocompromised individuals face a higher risk of developing any zoonosis (Chuang et al. 2008); due to the link between poverty and health, the most at-risk individuals may be the least informed about risks of zoonotic disease transmission (Steele and Mor 2015).

### Building climate resilience through decreasing inequity in companion animal fields

Racism, poverty, and other structural inequities are insipid and undermine the human–animal bond. Studies of racial disparities and companion animal welfare are sparse and primarily confined to the United States. Racial discrimination in housing and pet restrictions were also recently reported (Rose et al. 2020) and another study found that police shootings of dogs cluster in low-income communities of color (Bloch and Martínez 2020). A lack of racial diversity in US animal welfare volunteers has been reported (Neumann 2010) and the reasoning for underrepresentation of Black Americans in animal welfare has been explored via semi-structured interviews (Brown 2005). Social injustices are inextricably linked to climate change, and without immediate action will only worsen. Thus, we must get better at incorporating systemic social issues into our work. When we consider the intersection of social justice issues and companion animal science, an obvious starting place is animal sheltering. Animal sheltering and associated services touch the lives of most members in a community. Within its purview, animal services may provide necessary veterinary care for animals, reunite families with lost pets, enforce local, state, and federal laws concerning welfare and containment, and are side-by-side with first responders in a myriad of situations including during mental health crises (e.g., hoarding), paramedic emergencies (e.g., a car accident with a pet present), and in response to criminal activity (e.g., when a person’s pet is present during an apprehension in the community). Because of this breadth of work, there are ample opportunities to examine and improve procedures to ensure that these government agencies will serve all community members with equity and justice.

Though academics have been relatively quiet concerning social justice issues, it appears that awareness of inequity in companion animal sheltering has been increasing (e.g., Marceau 2019; Guenther 2020). The discussion has come mainly from activists in other fields (e.g., sociology, civil rights law) and from animal welfare nonprofit organizations (e.g., HASS 2021; PFL 2021), rather than researchers working in companion animal science. However, there is ample opportunity for scientists to incorporate climate justice studies into their existing work. For example, researchers who focus on shelter operations and adoption programs can include data on whether new or existing programs differentially affect vulnerable populations. Restrictive application processes involved in pet adoption—which often require potential adopters to live in a single-family house, have a backyard, allow a home visit to determine if the family is a “good fit,” or bring the entire family for a meet-and-greet—likely disproportionately affect Black, Indigenous, and People of Color. The subjective nature of home visits opens the door to racial and economic discrimination. Additional criteria such as excluding persons with a criminal history, persons who have previously relinquished a pet, or persons who do not have an established relationship with a veterinarian are even more opportunities for racial and socio-economic discrimination. Basic operating procedures, such as business hours and location, may also cater to particular racial, socio-economic, able-bodied, and age-specific demographics (Buley 2017). These are all understudied topics that could be incorporated into companion animal science research. Doing so would not only generate knowledge, but the data could be used to scientifically back policy change and create adaptation strategies for a more equitable future.

Even though working for social justice is certainly important for its own right, it also presents a crucial path to building resilient communities that can withstand climate change impacts. As climate change differentially impacts vulnerable communities, reducing inequity is a direct climate adaptation goal (IPCC 2014). As such, we encourage not just our fellow scientists, but all companion animal professionals—nonprofit advocacy organizations, government animal service agencies, private rescues, veterinarians, grant makers, dog trainers, pet store owners, educators, groomers, boarders, pet product companies, and anyone else who considers themselves a companion animal advocate—to seize this moment to be better. Incorporate anti-racist policies into every aspect of your work. Ensure that your staff are diverse and representative of your community. Commit to effective education of diversity, inclusion, and equity for all organization members. Examine your policies—written and unwritten—and find areas where you can be more equitable. Doing so is not only morally imperative, but it will undoubtedly serve to increase climate resiliency within our field.

## Companion animals contributing to climate change and exacerbating climate outcomes

Whereas previous sections draw links between the outcomes of climate change and its impacts on animals and the human–animal bond, it is also essential to highlight how companion animals, themselves, are contributing to the changing climate. The large estimates of companion animal populations worldwide suggest considerable environmental costs from natural resource use and pollution, most substantially so, from animal food production. Studies of GHG emissions and the ecological pawprint (EPP; a measure of the amount of productive land required) of companion animal food production consistently demonstrate pronounced environmental impacts and use of natural resources (Vale and Vale 2009; Rushforth and Moreau 2013; Okin 2017; Martens et al. 2019; Alexander et al. 2020). Globally, Alexander et al. (2020) estimate that pet food contributes 1.1–2.9% of global agriculture GHG emissions and ∼1% of global agricultural land use. The EPP and GHG emissions depend on a number of factors including the size and species being fed (larger species typically have a worse environmental impact), the meat sources used, and the energy systems used in food production. Premium cat food was estimated to have 3.3 times higher GHG emissions than market-premium food (Alexander et al. 2020). This means that a family who has multiple large dogs who are fed human-grade commercially-bought beef may present the most unsustainable pet ownership practices. In comparison, a family with pet rats, who may eat human leftovers, may present a more sustainable option based on such modeling.

Aside from feeding companion animals, other owner caretaking behaviors are also likely to have an environmental impact. For example, dog owners may use their personal car to bring their dog for exercise opportunities (e.g., walks in a natural area, dog parks, etc.; Mattioli et al. 2016). However, the transport sector accounts for more than a quarter of all energy use and produces substantial carbon dioxide emissions (IPCC 2014). Furthermore, waste—especially plastic waste—is a rapidly increasing threat to global health due to its resistance to decomposition (Andrady 2011). Most animal care products, including food, toys, waste disposal bags, and veterinary medical supplies are composed of single-use plastics. Since 1950, over 7,800 million metric tonnes of plastics have been produced—over half of which occurred since 2004 (Geyer et al. 2017). Plastic pollution environments can have damaging physical impacts, such as animal entanglement (Gall and Thompson 2015) and habitat destruction (Sheavly and Register 2007). Plastic pollution also has chemical effects, with toxicity effects bioaccumulating up the food chain (Worm et al. 2017). However, the impacts of companion animal care on contributing to plastic pollution is not well studied—albeit with the estimated 99 billion USD pet industry (APPA 2021) in the United States alone, it is likely that pet toys and supplies are probable contributors to waste pollution.

### Opportunities to mitigate impact of companion animals on climate change

Mitigation interventions to reduce the sources of GHG is an important objective as outlined in the Fifth Assessment Report of the IPCC (IPCC 2014). Human activities are the primary contributors to climate change, and a global focus on mitigating these impacts remains of utmost importance. However, considering the large, growing population of companion animals and their demonstrated environmental impacts, the need to implement mitigation strategies is also clear. “The Three R’s” that guide laboratory animal use in research to reduce animal suffering—replacement, reduction, and refinement (first conceived by Russell and Burch 1959; CCAC 2021) may also prove useful in environmental contexts. While some presented solutions may have varying feasibility depending on the individual and context, we present proposed solutions considering this replacement, refinement, and reduction framework for developing mitigation strategies. That being said, these suggestions are based on authors’ opinion and would greatly benefit from modeling strategies to identify the maximum reduction in emissions while safeguarding animal health and welfare and retaining benefits to companion animal owners.

Replacement refers to avoiding or replacing animal use when possible. Proposed replacement strategies to decrease the environmental impact of companion animal needs have been suggested by Vale and Vale (2009) including selecting smaller over larger dogs, selecting cats over dogs, and selecting companion animal species that are more sustainable. Alternative companion animals may be such that can utilize human food waste without significant health impacts (e.g., rats, pigs, etc.) or produce human food themselves (e.g., egg-laying hens).

Reduction refers to a strategy of fewer animals being used when possible. In the United States, ∼38% of households own dogs and 25% own cats, with an average of 1.6 dogs and 1.8 cats per household (AVMA 2021). Reduction of the number of animals kept as companions could reduce the EPP of companion animal care (Martens et al. 2019). Reduction strategies may include sharing ownership of animals across multiple families, adopting existing animals rather than breeding new animals, and having temporary ownership such as fostering or pet sitting arrangements. Programs that target reducing free ranging populations, where harmful to people or the environment, may also be considered within this strategy. For example, humane Trap-Neuter-Vaccinate-Return programs for feral cats have been successful in certain contexts to reduce overall numbers of community cats that may be harmful to biodiversity (c.f. Crawford et al. 2019; Schaffner et al. 2019).

Finally, refinement includes any modifications to animal care or procedures that can minimize negative impacts. Refinements in feeding animals may include a consideration of pet food ingredients as well as the quantity of food fed. Owners overfeeding their pets also have a negative impact on both the environment (Rushforth and Moreau 2013; Swanson et al. 2013; Schwartz 2014; Martens et al. 2019) and pet health. Replacement of high GHG emitting and high EPP meat sources (such as beef) in companion animal food with other sources such as poultry and fish (Vale and Vale 2009; Schwartz 2014), lab-grown meat equivalents (Su et al. 2018), insect-based protein (Han et al. 2017), or plant-based sources in diets have been considered (Pimentel and Pimentel 2003; Reijnders and Soret 2003; Wirsenius et al. 2010; Okin 2017). Alternative diets such as a vegetarian or insect-based diets may be a more environmentally conscious food source for companion animal diets (Knight and Leitsberger 2016 ; Halloran et al. 2017). Furthermore, utilizing by-products that are not suitable for human consumption may significantly reduce the EPP (Alexander et al. 2020). Refinement in this context may include increasing bioavailability and digestibility of food (Swanson et al. 2013) or processing human food waste into companion animal food (Rastogi 2010; Castrica et al. 2018). In fact, free ranging dogs utilize both human food waste as well as human excrement effectively (Butler et al. 2018), so reversal of the modern approach of using commercial dog food may be additionally considered. However, understanding the complete environmental impacts of food production and the determination of associated species-specific nutritional adequacy is needed. Further refinements, which may include reducing and replacing plastic toys and waste disposal bags, could be other simple changes to decrease environmental damage. Strategies to reduce predation by owned cats include restricting or managing outdoor access, wearable predation prevention devices such as specialized collars and bells, and nutrition and activity interventions (Cecchetti et al. 2020). However, developing humane cat predation prevention procedures while simultaneously meeting cat welfare needs remains an urgent need in both feline and climate research. Finally, appropriate selection of a companion animal to match the owner’s current climate may reduce impacts. For example, purchasing a long-coated dog with cold climate origins in a hot climate will likely result in the owner providing additional air conditioning in the home, trips to a groomer, and using a personal vehicle for safe exercise opportunities—all of which will likely have negative environmental impacts.

Overall, considering the growing population of companion animals worldwide and the resulting widespread use of natural resources, developing strategies for both individuals and industries to mitigate the effect of companion animals is vital. Furthermore, gaining a better understanding of feasibility and impact of mitigation strategies is needed. It is possible, for example, that switching from one pet food to another may reduce EPP, but is this reduction negligible or meaningful on a global scale (see discussion in Alexander et al. 2020)? Likewise, companion animal ownership and caretaking behaviors must be considered in the full array of human activity. When considering overall harms and benefits of companion animal ownership, it is important to consider whether pet ownership itself improves pro-environmental behaviors in society. Attachment to pets positively influences children’s later attitudes toward animals (Paul and Serpell 1993; Hawkins and Williams 2017), but the relationship between pet ownership in childhood and later concerns for environmental issues is less clear (Paul and Serpell 1993). However, pet-owning children that practiced more pet-caring behaviors were also more environmentally concerned (Torkar et al. 2020). Likewise, having a high attachment to the pet, rather than being an owner, was a determinant of subsequent vegetarian or vegan diets in adulthood (Rothgerber and Mican 2014). Pet owners, generally, are more supportive of wildlife management strategies that prevent species extinction, but are also opposed to wildlife conservation strategies that restrict pet ownership or associated behaviors (Shuttlewood et al. 2016)—such as controlling free roaming cat populations (Grayson et al. 2002; McDonald et al. 2015) and leashing dogs (Williams et al. 2009). Similarly, while dog owners tend to walk more (Christian et al. 2018), they also tend to rely on private cars for getting to the exercise location (Kent and Mulley 2017). Finally, whereas popular media frequently suggests that pets are child substitutes and may lead to a decreased human population, research has not found a difference in the number of children by pet ownership (Saunders et al. 2017); in fact, having a child positively predicted dog ownership (Westgarth et al. 2007). Ultimately, more research is needed to better understand whether there is a causal link between pet ownership and environmentally-conscious behaviors. These questions further highlight the importance of systems thinking in the creation of mitigation and adaptation plans in our field.

## Considerations for constructing mitigation and adaptations strategies

As we work to identify mitigation or adaptation strategies for our field, we must keep several things in mind - our plans must be (1) equitable to protect human wellbeing (Shi et al. 2016), (2) sustainable and not contribute to the worsening of the climate (Eriksen et al. 2011), (3) respect animal health, welfare, and agency (Shields and Orme-Evans 2015), and (4) measurable (Haasnoot et al. 2018; Olazabal et al. 2019). These requirements may present unique challenges and further highlight the need for systems thinking.

Because the human–animal bond is profound (Walsh 2009a, 2009b), mitigation and adaptation plans must aim to keep owners and their animals together. When considering plans involving companion animals, their considerable value in our lives must be recognized. An example of a plan that would not pass the mark may be programs to eradicate free ranging populations of urban animals, such as dogs. Whereas reducing populations of urban animals may ameliorate concerns of zoonotic diseases, these programs must be implemented only with the backing of the target communities. A devastating example of a harmful approach is capturing and rehoming free ranging dogs in Indigenous communities in Canada. This has been done repeatedly and without permission, with “rescuers” unaware or indifferent to the reality that these dogs were community-owned, cared for, and culturally important. This blatant stealing caused direct harm to owners and sabotaged working relationships between Indigenous communities and other animal welfare advocates (Bressette 2020).

The integration of indigenous or traditional knowledge has been identified as a necessary inclusion into successful, feasible, and ethical adaptation plans (Riedlinger and Berkes 2001; Gyampoh et al. 2009; Leonard et al. 2013). Committing to climate justice in companion animal science and welfare may take on many forms. It necessitates not only that we adapt our research goals, but also that we modify all aspects of our professional duties such as inclusive hiring, partnering with indigenous leaders, and re-examining our philosophies and procedures to ensure alignment with anti-racist and anti-colonial teaching.

Adaptation strategies must strive to not negatively impact the environment and thus, not contribute to the changing climate. Fear of emerging zoonotic disease may increase the use of antibiotics and thus protect the health of human and nonhuman animals in the short-term but is also likely to significantly harm long-term public health through increasing prevalence of antimicrobial resistance (AMR)—an example of an unsustainable plan of action. Plans must also respect animal health and welfare. For example, the promotion of alternative diets, such as vegetarian diets, by companion animals may be beneficial in GHG emissions, but may also present animal health risks (e.g., Kanakubo et al. 2015). This presents an opportunity for collaboration between researchers in animal health, veterinarians, and climate scientists, given the veterinary field’s expressed interest, but perceived lack of opportunity, to address climate change in their work (Kramer et al. 2020; Pollard et al. 2020).

A final, crucial aspect of an adaptation plan is that the impact is measurable (Haasnoot et al. 2018; Olazabal et al. 2019). The hypothesized increase in community resilience following the implementation of the plan must be measured and reported, and if insufficient progress is demonstrated, amended. Social and climate scientists designed several high-level approaches that may be utilized to create adaptation plans, but all plans tell us to focus on immediate identified needs and amend when new needs or deficiencies are discovered (Haasnoot et al. 2018). Thus, we do not need to succumb to inaction caused by future uncertainties of climate change and the overwhelming amount of work that needs to be done, but instead focus on what is just in front of us and needs doing.

## Conclusion

Climate change has been recognized as the most urgent matter impacting human society. Companion animals impact and are impacted by the changing climate through their intrinsically linked relationships with people. And, companion animal scientists, who are often generalists and systems thinkers, are in an ideal position to address these impacts. As examples, we identified several climate change outcomes, such as warming temperature, migration of people, increases in disasters, crises, and human inequities, and attempted to connect these outcomes to already identified and hypothesized impacts on companion animals and the human–animal relationship. We also summarized how companion animals and owners’ caretaking behaviors are impacting climate change through the use of finite natural resources as well as pollution and carbon emissions. We end on some considerations on creating mitigation and adaptation plans with a focus on equity, sustainability, respect for animal health and welfare, and measurability as key features.

The stark reality is that our climate is changing and it is affecting every aspect of our lives, including our science, our study populations, and our communities. As scientists and global citizens, we must incorporate climate change mitigation and adaptation strategies into our work, even if we are not in the climate science field. We hope that in a near future, much more research on the relevance of companion animals to climate change will be conducted allowing us to clarify links—reducing hypothetical assertions and providing clear directions to our communities. We hope that this is the beginning of a new, necessary conversation, and that as a field we can learn and grow together for a more just and resilient future.

## Authors’ contribution 

The idea for this work was conceived by A.P. All authors contributed equally to the writing and editing of this manuscript.
